# Anxiety and amygdala connectivity during movie-watching

**DOI:** 10.1016/j.neuropsychologia.2022.108194

**Published:** 2022-05-03

**Authors:** Peter A. Kirk, Oliver J. Robinson, Jeremy I. Skipper

**Affiliations:** a UCL Institute of Cognitive Neuroscience, UK; b UCL Experimental Psychology, UK; c UCL Clinical, Educational and Health Psychology, UK

**Keywords:** Naturalistic, fMRI, Anxiety

## Abstract

Rodent and human studies have implicated an amygdala-prefrontal circuit during threat processing. One possibility is that while amygdala activity underlies core features of anxiety (e.g. detection of salient information), prefrontal cortices (i.e. dorsomedial prefrontal/anterior cingulate cortex) entrain its responsiveness. To date, this has been established in tightly controlled paradigms (predominantly using static face perception tasks) but has not been extended to more naturalistic settings. Consequently, using ‘movie fMRI’—in which participants watch ecologically-rich movie stimuli rather than constrained cognitive tasks—we sought to test whether individual differences in anxiety correlate with the degree of face-dependent amygdala-prefrontal coupling in two independent samples. Analyses suggested increased face-dependent superior parietal activation and decreased speech-dependent auditory cortex activation as a function of anxiety. However, we failed to find evidence for anxiety-dependent connectivity, neither in our stimulus-dependent or -independent analyses. Our findings suggest that work using experimentally constrained tasks may not replicate in more ecologically valid settings and, moreover, highlight the importance of testing the generalizability of neuroimaging findings outside of the original context.

## Highlights

• Using ‘movie fMRI’, we tested whether trait anxiety correlates with face-dependent amygdala-prefrontal coupling.
• We observed altered superior parietal activation to faces and auditory cortex activation to speech as a function of anxiety.
• We failed to find evidence for anxiety-dependent amygdala-dmPFC connectivity in stimulus-dependent or -independent analyses.
• Our findings highlight the importance of testing the generalizability of neuroimaging findings outside of the original context.

## 1 Introduction

Anxiety bears a powerful impact on public ill health (Kessler et al., 2005). As such, it is often understood as a mental disorder. However, anxiety is also a ubiquitous, healthy emotional response to anticipated threats. A breadth of research has investigated the biopsychological mechanisms underpinning pathological anxiety, but relatively less work has focussed on this more normative, adaptive manifestation. Our current understanding of anxiety is thus fairly limited. By conducting studies with healthy humans, we can inform models of core threat circuitry (Robinson et al., 2012). As both forms of anxiety appear to demonstrate considerable functional convergence in the brain (Chavanne and Robinson, 2020), studies of adaptive anxiety also hold potential to accelerate discovery in the pathological domain by helping researchers generate clinically-relevant hypotheses and tools for treatment evaluation (Grillon et al., 2019).

A key function of anxiety is to promote vigilance toward potential threats in the environment, but chronic engagement of this system may underlie pathology (Robinson et al., 2012). Research has reliably shown anxiety biases the processing of faces (Surcinelli et al., 2006; Robinson et al., 2011), a highly salient feature of the environment for (highly social) humans. Consequently, neuroimaging experiments of anxiety have predominantly utilized face-perception tasks, often focussing on amygdala activation. There are numerous studies demonstrating increases in amygdala response to faces parametrically scales with affective bias (i.e. fear/anxiety; Killgore and Yurgelun-Todd, 2005, de Groot et al., 2021; Somerville et al., 2004) and is seen in the presence of anxiety disorders (Cooney et al., 2006). Subsequent research has demonstrated, however, that within-subject amygdala response across time holds moderate-to-poor reliability (Nord et al., 2017; Sauder et al., 2013). Taking a modular, amygdala-centric view may indeed be over-simplistic, and unable to sufficiently capture biological dynamics underlying anxiety. Instead, a more holistic explanation may come from studying the wider circuitry associated with the amygdala.

There is now substantial evidence from the animal literature implicating amygdala-prefrontal circuitry in threat processing (for a review, see Robinson et al., 2019), wherein dorsomedial prefrontal/anterior cingulate cortex (dmPFC/ACC) provides top-down entrainment of amygdala reactivity, and this bears importance for responding to potential threat (Karalis et al., 2016). Recruitment of this circuit has also been demonstrated in human subjects: increased amygdala-dmPFC/ACC coupling during the processing of fearful faces has been demonstrated in humans undergoing induced anxiety (Robinson et al., 2012). Notably, this coupling positively correlates with self-report measures of anxiety symptoms and may constitute a more temporally stable signal than amygdala reactivity alone (Nord et al., 2019). This circuitry is posited to drive anxiety-induced amplification of salient stimuli (Robinson et al., 2012). Thus, excessive recruitment of this circuitry could result in chronic attentional biases for threat (Robinson et al., 2012). The implication of this ‘aversive amplification’ circuit in humans has been replicated elsewhere, such as in: clinical samples (Demenescu et al., 2013; Robinson et al., 2014), stimulus-independent analyses (Vytal et al., 2014), emotion regulation tasks (Zotev et al., 2013), and predator-prey paradigms (Gold et al., 2015). Of course, other fMRI paradigms have demonstrated anxiety-dependent amygdala connectivity to regions such as ventromedial prefrontal cortex (Kim et al., 2011) and insula (Roy et al., 2013). Nonetheless, increased amygdala-dmPFC/ACC coupling is a consistent finding, and as such, is a commonly adopted model for biomarker-focused anxiety research (Brehl et al., 2020; Grillon et al., 2019; Yuan et al., 2016).

Despite a multitude of fMRI studies investigating the neural substrates of anxiety, a methodological gap remains in the literature. Research has predominantly relied on static, unnatural face stimuli presented without any context. These paradigms deviate from the natural perception of faces in day-to-day settings (Barrett et al., 2007) and may lead to misclassification of expressions, particularly those of fearful/sad faces (Carlisi et al., 2020). Such tightly-controlled experiments could lead to theory that may overlook dynamic, context-dependent networks in the brain (Skipper, 2014; Sonkusare et al., 2019; Spiers and Maguire, 2008). Previous studies have built a fundamental understanding of core threat circuitry, but whether anxiety-related brain activity in less constrained settings can be explained by current theory has yet to be established.

The recent uptake in ‘movie fMRI’ paradigms—where participants watch real movies whilst in the fMRI scanner—allows the opportunity to address some of these concerns. This method may help validate and extend current models of anxiety, improve data quality, and inform biomarker-based research (Eickhoff et al., 2020; Hasson et al., 2010; Finn and Bandettini, 2020; Vanderwal et al., 2009). Indeed, two studies so far have demonstrated *within-subject* amygdala-prefrontal coupling during anxiety-inducing movie scenes (Hudson et al., 2020; Kinreich et al., 2011). To our knowledge however, there exists no study investigating whether *between-subject* differences (i.e. self-reported symptoms of anxiety) in amygdala-prefrontal circuitry are seen in ecologically-richer contexts. Therefore, in the present preregistered two-experiment study, we investigated the relationship between self-reported anxiety and amygdala-connectivity in two independent movie-watching fMRI datasets.

### 1.1 Database summary

**Fig. 1:**
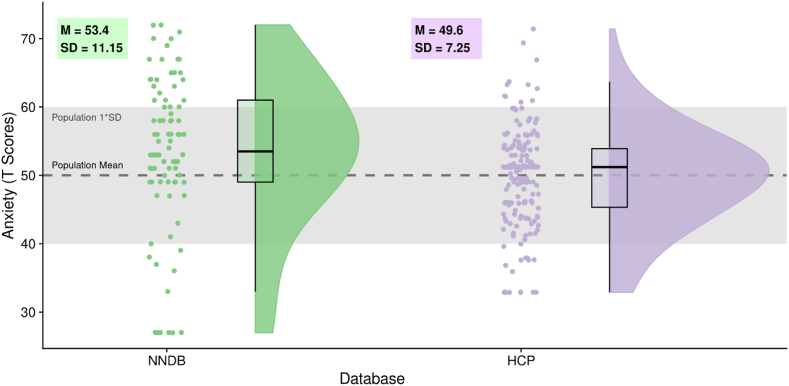
Raincloud Plots (Allen et al., 2019) of anxiety scores for the Naturalistic Neuroimaging Database (NNDB) and Human Connectome Project (HCP): jittered data points represent individual participants, box plot hinges mark 25th/50th/75th percentiles, box whiskers indicate 1.5*interquartile range, and density plots represent smoothed distribution.

In the present project, we used two openly available databases which include movie fMRI, the Naturalistic Neuroimaging Database (Aliko et al., 2020; experiment 1) and Human Connectome Project (Van Essen et al., 2013; experiment 2). A table describing participants and fMRI sequences is provided for comparisons (Table 1). Both databases required participants to have no history of psychiatric or neurological illness. This information is elaborated on within experiment-specific reporting. Distributions of anxiety scores (from the NIH Toolbox's Fear-Affect CAT Age 18+; NIH Toolbox, n.d.) are also provided (Fig. 1).

**Table 1:** Key cross-experiment comparisons. Columns (left to right) refer to: databases used; participant N (including gender and age-range); MRI magnet strength; repetition time; echo time; flip angle; voxel size; multiband acceleration; phase encoding direction.

Database	N	Magnet	TR	TE	FA	Voxels	MB	Phase
NNDB	86 (42 F/44 M; 18–58 years)	1.5 T	1000 ms	54.8 ms	75°	3.2 mm^3^	4	A- > P
HCP	178 (108 F/70 M; 22–31+ years)	7 T	1000 ms	22.2 ms	45°	1.6 mm^3^	5	Variable

## 2 Hypotheses

### 2.1 Naturalistic neuroimaging database (experiment 1)

Based on the ‘aversive amplification’ circuitry hypothesis (Robinson et al., 2014), we preregistered the following predictions in regard to our analyses of the Naturalistic Neuroimaging Database:

1. Self-reported symptoms of anxiety will positively correlate with face-dependent dmPFC-left amygdala functional connectivity.
2. Self-reported symptoms of anxiety will positively correlate with face-dependent dmPFC-right amygdala functional connectivity.

### 2.2 Human Connectome Project (experiment 2)

Prior to reanalysis in the updated naturalistic neuroimaging database (see *Method*), we previously observed depleted amygdala-cingulate and -middle frontal gyrus connectivity as a function of anxiety (reported below). As such, we hypothesized a similar effect on an independent dataset to provide out-of-sample validation. Specifically, we predicted:

1. Self-reported symptoms of anxiety will negatively correlate with stimulus-independent amygdala-dmPFC functional connectivity during movie-watching.
2. Self-reported symptoms of anxiety will negatively correlate with stimulus-independent amygdala-middle frontal gyrus functional connectivity during movie-watching.

## 3 Method

### 3.1 Preregistration

Our planned analyses were preregistered. Along with our code, these are available on the Open Science Foundation (https://osf.io/345nj/).

### 3.2 Naturalistic neuroimaging database (experiment 1)

We conducted analyses on the Naturalistic Neuroimaging Database (Aliko et al., 2020). In brief, the database contains a set of 86 right-handed participants (42 females; aged 18–58 years, *M* = 26.81, *SD* = 10.09) viewing entire movies whilst under functional MRI. Participants watched one movie during scanning, and the movie varied between participants (10 movies in total; minimum length = 92 min; maximum length = 148 min; Table 2). Scanning was conducted on a 1.5 T Siemens MAGNETOM Avanto (T2*-weighted images: TR = 1000 ms, TE = 54.8 ms, Slices = 40; FA = 75°, Voxel size = 3.2 mm^3^, MB = 4). The functional data had already been preprocessed using the following steps: slice-time correction; volume registration; registration of functional images to warped anatomical scan; spatial smoothing to 6 mm FWHM; normalization; and manual ICA artifact rejection. The use of ICA-denoising is particularly relevant to our analyses in addressing physiological confounds (e.g. respiration) that would otherwise be of relevant concern (Chang and Glover, 2009; Glasser et al., 2019). For a full overview of database details, see Aliko et al. (2020).

Since preregistering our analyses, the naturalistic neuroimaging database released a new version (v2.0), which contains a fix for an issue with timeseries scaling for runs of different lengths as well as the implementation of a standardized preprocessing pipeline, ‘afni_proc.py’. In the present manuscript, we summarize our original findings under results and report the updated analyses. The full reporting of our original results can be found in our preprint (https://psyarxiv.com/aumk3, version 1).

#### 3.2.1 Stimulus onsets

**Fig. 2:**
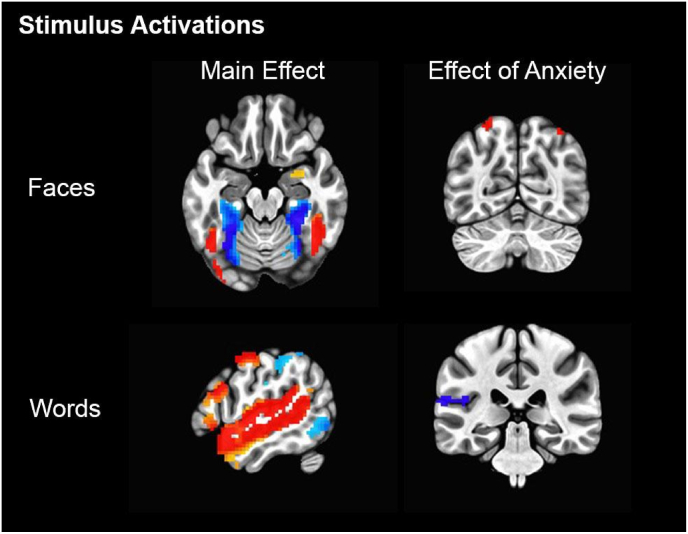
Whole-brain results (*p*_uncorr._<0.001, cluster-corrected at k ≥ 11; red voxels = increased activation, blue voxels = reduced activation) demonstrating brain activations to faces and words and how activation to these stimuli correlate with self-reported anxiety. (For interpretation of the references to colour in this figure legend, the reader is referred to the Web version of this article.)

Word onset and face data from the movies was extracted using Amazon Web Services’ *Transcribe* (https://aws.amazon.com/transcribe/) and *Rekognition* (Amazon, n.d.). Detected face and word onsets had an associated confidence score for being correct (0–100%). For this, we selected an arbitrary threshold of 90%. Across movies, an average of 92.7% (*SD* = 2.63%) of faces detected fell within this threshold (Table 2). *Rekognition* has been shown to perform well in naturalistic detection of faces (Hsu and Chen, 2017). *Transcribe* word information was matched and aligned with subtitle information (see Aliko et al., 2020). To further validate the accuracy of the face and word detection algorithms, we specified confirmatory contrasts, wherein we saw expected fusiform and auditory cortex activation respectively (Fig. 2). Face and word onsets had variable durations. For the purposes of obtaining psychophysiological interaction terms, onsets were resampled into stable 200 ms windows (5 Hz).

**Table 2:** Naturalistic Neuroimaging Database summary: movie watched, number of subjects, movie length, and the proportion of detected faces that fell within our confidence threshold.

Movie	N	Duration (mins)	Proportion of faces over 90% confidence
500 Days of Summer	20	91.17	95%
Citizenfour	18	113.40	94%
12 Years a Slave	6	128.53	91%
Back to the Future	6	111.23	92%
Little Miss Sunshine	6	98.33	91%
The Prestige	6	125.25	91%
Pulp Fiction	6	148.03	91%
The Shawshank Redemption	6	136.35	89%
Split	6	112.32	97%
The Usual Suspects	6	101.70	96%

#### 3.2.2 Behavioural data

Approximately two weeks prior to scanning, participants completed questionnaires from the NIH Toolbox (NIH Toolbox, n.d.). Of relevance to the present study, this included an emotion battery (Salsman et al., 2013). Here, we used the *Fear-Affect CAT Age 18+* uncorrected T-scores. The questionnaire measures “symptoms of anxiety that reflect autonomic arousal and perceptions of threat” (NIH Toolbox, n. d). This holds convergent validity with other, commonly used anxiety questionnaires (Salsman et al., 2013; Schalet et al., 2014).

### 3.3 Human Connectome Project 7 T dataset (experiment 2)

The Human Connectome Project is a large-scale database of multimodal MRI data (Van Essen et al., 2013). Within the database is a subset of functional scans (N = 184; runs = 4) collected with a 7 T Siemens MAGNETOM whilst participants watched movie scenes across 4 sessions/2 days. Participants watched 14 movie clips (duration range = 65–255s) interspersed with 22 rests (20s) and 4 repeated video validation clips (83s). We provide a summary below (Table 3; for full details, see https://protocols.humanconnectome.org/HCP/7T/). This constituted the dataset for experiment 2. Six subjects had at least 1 run of movie data missing. These were excluded, leaving a final N = 178. Subject's specific ages are not provided in this database, rather.

**Table 3:** Presentation order for Human Connectome Project movie-watching data. Rests were 20s and validation clips were 83s.

Day 1		Day 2	
Block 1	Block 2	Block 3	Block 4
Rest	Rest	Rest	Rest
Two Men (244s)	Inception (227s)	Off The Shelf (181s)	Home Alone (232s)
Rest	Rest	Rest	Rest
Welcome To Bridgeville (222s)	Social Network (259s)	1212 (185s)	Erin Brockovich (230s)
Rest	Rest	Rest	Rest
Pockets (188s)	Ocean's Eleven (249s)	Mrs. Meyer's Clean Day (204s)	The Empire Strikes Back (255s)
Rest	Rest	Rest	Rest
Inside The Human Body (64s)	Validation clip	Northwest Passage (143s)	Validation clip
Rest	Rest	Rest	Rest
Validation clip		Validation clip	
Rest		Rest	

#### 3.3.1 Preprocessing

The data available was already preprocessed using a minimal pipeline (*fMRIVolume*: gradient-distortion correction, FLIRT-based motion correction, TOPUP-based unwarping, coregistration, transformation to MNI, intensity normalization & bias field removal; Glasser et al., 2013). In addition to these steps, we smoothed the data to 6 mm FWHM (’3dBlurToFWHM’; masked in subject-specific grey matter) to match the smoothness of the data in experiment 1. Key differences to the preprocessing performed on the Naturalistic Neuroimaging Database were: the use of TOPUP-based unwarping; lack of ICA-denoising (in volume-based data); and lack of slice-time correction.

#### 3.3.2 Behavioural/demographic data

The Human Connectome Project also used the NIH Toolbox, and thus contains the *Fear-affect CAT 18+* uncorrected T-scores which we used for our analyses. Human Connectome Project age data is provided in pseudonymized brackets (22–25; 26–30; 31–35; 36+). For the purposes of our regressions, these were coded as categorical factors. As certain age by gender cells did not have sufficient N to run our group-level model (relevant N's: 22–25 years females = 1; 36+ years females = 2; 36+ years males = 0), ages were re-coded into two brackets (22–30 years [M = 56; F = 51]; 31+ years [M = 14; F = 57]).

### 3.4 Analyses

fMRI time series extraction and modelling were conducted in AFNI (Cox, 1996) on an Ubuntu 18.04 OS (GNU Bash). Relevant AFNI functions are denoted in parentheses. Due to memory constraints, within-subjects analyses were conducted on sections of slices at a time (’3dZcutup’). Beta-weight outputs were then concatenated back into whole-brain maps (’3dZcat’) before group-level analysis. All analyses used two-sided tests thresholded at α = .05.

#### 3.4.1 Regions of interest masks

Our key regions of interest include the amygdala and dorsomedial prefrontal cortex (dmPFC). Our amygdala ROIs were selected through individual anatomical parcellations of T1 images in Freesurfer (Fischl, 2012). ROI masks were visually inspected for successful segmentation. Our dmPFC ROI was a functional mask from a recent meta-analysis of anxiety (Chavanne and Robinson, 2020; ‘patients > controls 20 mm’ at ∼[0 25 40]).

#### 3.4.2 Naturalistic neuroimaging database models

Our within-subjects models were constructed using generalized psychophysiological interactions (McLaren et al., 2012). This enabled us to test context-dependent connectivity with amygdala above and beyond task-related activation and covariation with the raw amygdala time series. In line with AFNI recommendations (https://afni.nimh.nih.gov/CD-CorrAna), we conducted the following preparatory pipeline for each subject: 1) extract time series of amygdala (’3dmaskave’); 2) upsample to resolution of stimuli onsets (’1dUpsample’); 3) deconvolution of seed time series (‘waver’ [basis function = BLOCK], then ‘3dTfitter’); 4) obtain and convolve interaction terms for stimuli onsets (’1deval’, then ‘waver’ [basis function = BLOCK]); and 5) downsample interaction terms to resolution of TR (’1dcat’). We built our 1st level design matrices (’3dDeconvolve’, -mask “sub-*_T1w_mask”) inputting 9 regressors: face onsets convolved with a hemodynamic response function (HRF) [basis function = dmBLOCK], HRF-convolved word onsets [basis function = dmBLOCK], left amygdala seed time series, right amygdala seed time series, left amygdala face interaction term, right amygdala face interaction term, left amygdala word interaction term, right amygdala word interaction term, and a constant [-polort 0].

We constructed a group-level matrix using AFNIs multivariate modelling (’3dMVM’) with 1st-level beta-weight maps inserted as within-subject variables (’-wsVars’). Anxiety, gender, age, and movie watched were inputted as between-subject regressors (’-bsVars’). The inclusion of the latter regressors in our model allowed us to test differences above and beyond those induced (linearly) by specific movies, age, and/or gender. All analyses were coded as general linear tests (’-gltCode’). Our whole-brain analyses used t tests with an initial cluster-defining threshold of *p*_uncorr._<0.001 before whole-brain cluster correction (’3dFWHMx’ with group residuals, ‘3dClustSim’; i.e. k ≥ 10.4). 3dMVM and 3dClustSim were constrained using subject-wide averaged masks (“sub-*_T1w_mask”; ‘3dMean’). Whole-brain results are reported in MNI space.

Given that our hypotheses sought to test a specific functional landmark within the medial prefrontal cortex, whole-brain statistical correction could have been overly conservative. As such, we also conducted ANCOVAs of dmPFC-averaged betas for our main hypothesis-testing in the Naturalistic Neuroimaging Database. Following beta-weight extraction, analysis of dmFPC ROIs were conducted for our main hypothesis-testing in JASP (JASP Team, 2020). We supplemented these analyses with Bayesian equivalents using JASP's default, non-informative priors. Bayes Factors are reported as evidence for the null (BF_01_). Winning models in the Bayesian ANCOVAs were those with the highest BF_01_ relative to the null (intercept only model). The relative predictability was calculated by dividing Bayes Factors between models.

#### 3.4.3 Human Connectome Project models

We first removed effects of no interest from our raw time series for each run using ‘3dDeconvolve’ by including baseline terms with drift [-polort A] and 12 motion parameters (raw + temporal derivatives) as regressors to produce a cleaned, error time series. We then extracted amygdala seeds (’3dmaskave’) from the cleaned time series before computing left and right amygdala-whole brain beta-weights and correlation maps (’3dDeconvolve’; ‘3dcalc’; r maps were Fisher z-transformed). Volumes which included majority rest or validation clips (i.e. assigning 0 to TRs in seed regressors). The first 10 s of movie volumes were also excluded to rule out influence from rests.

We took within-subjects amygdala time series beta-weight maps and whole-brain correlations forward to a group-level model (’3dMVM Chen et al., 2014) with anxiety scores, age, gender, and run as regressors. This was again masked with subject-wide average grey matter. Whole-brain analyses employed cluster-level correction (’3dClustSim’) using a spatial autocorrelation function estimated from group-level residuals (’3dFWHMx’). We inspected results using contrast-specific two-sided t tests at two levels of voxelwise correction: *p*_uncorr._<0.01, and *p*_uncorr._<0.05, which resulted in cluster thresholds of k ≥ 431.7 and k ≥ 1913.7 respectively.

For post-hoc exploratory-testing, we also made use of a canonical 400 parcel-level segmentation (Schaefer et al., 2018). Linear models including anxiety scores, age, and gender and regressors were conducted for each movie clip (14) by amygdala connection (2) by parcel (400) combination (total = 11,200). Beyond this, we did not submit these to formal hypothesis-testing; rather, we visualized amygdala connectivity x anxiety t scores on a per clip basis to aid in interpretation of our results.

#### 3.4.4 Control analyses

We additionally included post-hoc control analyses to test whether connectivity results were driven by anxiety-correlated noise across both datasets. We reconducted our analyses using calcarine sulcus as a seed (instead of amygdala). This was to test whether any of our anxiety-dependent psychophysiological or seed results may be a product of global signal correlations, rather than an effect specific to amygdala connectivity.

### 3.5 Deviations from preregistration

We note the following deviations from preregistration for the Naturalistic Neuroimaging Database:

• We did not preregister a plan to handle centering for the purposes of our group-level intercepts. Anxiety scores were mean-centered. As age showed a strong positive skew (supplemental 1), this was median-centered for the purposes of group-level intercepts.
• We preregistered to construct multiple group-level models using F tests. However, we streamlined this by having a single coherent group-level model, coding two-sided t tests for planned analyses whilst retaining the same statistical thresholding. This was done to provide directionality (e.g. increased vs decreased connectivity).
• We decided to re-inspect our results at more liberal voxelwise thresholds in order to investigate relatively more diffuse effects (Cox, 2019). We also included a word by anxiety correlation in the analysis. Post-hoc tests are noted within text.
• As the intersection of between-subjects grey matter resulted in an overly thin mask, we changed required overlap from 100% to 95% of participants.
• In the present manuscript, we summarize our original findings and report the updated analyses for version 2 of the naturalistic neuroimaging database. The full reporting of our original results can be found in our preprint (https://psyarxiv.com/aumk3, version 1).
• The control analysis was conducted post-hoc.

We note the following deviations from preregistration for the Human Connectome Project dataset:

• We preregistered to code ages into four categories. However, as certain gender by age cells did not have sufficient N to run our model, we collapsed ages into two categories (see *behavioural/demographic data*).
• As the intersection of between-subjects grey matter resulted in an overly thin mask (supplemental 3), we changed required overlap from 100% to 95% of participants.
• ROI and control analyses were conducted post-hoc.

## 4 Results

### 4.1 V1 results (naturalistic neuroimaging database)

We originally conducted our analyses on the naturalistic neuroimaging database using an earlier version of the dataset (NNDb V1). As our updated analyses (on NNDb V2) altered our inference, we report here a brief summary of the relevant original findings. Firstly, we saw no correlations with anxiety scores for face onsets. For our hypothesized *stimulus-dependent* connectivity analyses, we did not observe any correlations between psychophysiological interactions and anxiety scores. We did not observe effects of anxiety on *stimulus-independent* connectivity measures at our initial voxelwise threshold. We then re-inspected results with more liberal voxelwise thresholding (*p* < .01, *p* < .05; cluster-corrected). For our seed regressors, we observed correlations between anxiety and: right amygdala-anterior/mid cingulate (voxelwise p < .05, peak = [1 43 13], 273 voxels) and left amygdala-right anterior middle frontal gyrus connectivity (voxelwise p.<0.05, peak = [31 58 22], 175 voxels; lateral Brodmann area 10). Contrasting these results with main effects suggested these were functionally excitatory connections independent of faces/words, but were depleted as a function of anxiety.

### 4.2 Activation-based analyses (naturalistic neuroimaging database)

To provide a basic characterisation of the narutalistic neuroimaging dataset and validate the use of our onset regressors, we ran two-tailed t tests for altered activation to 1) faces and 2) words. As expected, we saw increased activation to faces in fusiform gyri (peak = [40–89 -17], 1133 voxels), notably overlapping with meta-analytic fusiform face area activation (peaks = [39–53 -22; -40 -54 -23]; Aliko et al., 2020; Berman et al., 2010). We did observe separate clusters of reduced lingual/fusiform gyri activation to faces (left peak = [−29 -50 -8], 634 voxels; right peak = [28–56 -8], 676 voxels), though these were more distal to typical face-selective activation. In regard to word onsets, we saw increased activation in primary auditory cortices/superior temporal gyrus (left peak = [−68 -11 4], 2092 voxels; right peak = [67–5 1], 1379 voxels; Fig. 2).

We saw two cluster-corrected positive correlations with anxiety scores for faces in superior parietal lobe (left peak = [−29 -68 64], 18 voxels; right peak = [34–65 58], 23 voxels). For our post-hoc word onset analysis, we observed a cluster in left auditory cortex to negatively correlate with anxiety scores (peak = [−65 -32 16], 34 voxels).

### 4.3 Face-dependent amygdala connectivity (naturalistic neuroimaging database)

**Fig. 3:**
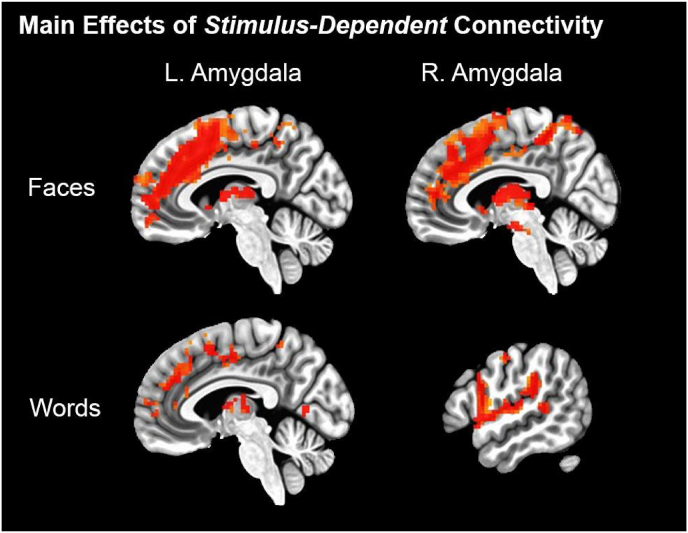
Whole-brain results (*p*_uncorr._<0.001, cluster-corrected at k ≥ 11) demonstrating increased amygdala connectivity as a function of faces and spoken words.

For PPI main effects, we observed increased connectivity as a function of faces, notably increased connectivity between amygdala and bilateral medial prefrontal/anterior cingulate cortex (Fig. 3). We also observed effects of increased amygdala connectivity as a function of words in medial prefrontal cortex (left and right amygdala terms) and auditory cortex/superior temporal gyrus (right amygdala only).

For our hypothesized connectivity analyses, we did not observe any cluster-corrected correlations with anxiety scores. As whole-brain statistical correction could be overly conservative, we conducted ROI analyses to test our hypotheses. Congruent with the whole-brain tests, ROI ANCOVAs also failed to demonstrate a significant effect of anxiety (full reporting in supplemental 2). We repeated analyses post-hoc with more liberal voxelwise threshold (*p* < .01 & *p* < .05; cluster-correction thresholds = 35.8 & 144.9 respectively. We observed a positive correlation between anxiety scores and face-dependent amygdala-superior temporal sulcus connectivity (voxelwise *p* < .05; left peak = [−68 -11 4], 250 voxels; right peak = [61–20 16], 223 voxels).

### 4.4 Stimulus-independent analyses

#### 4.4.1 Naturalistic neuroimaging database

We conducted two preregistered, exploratory left and right amygdala seed-whole brain correlations. This tested for effects of amygdala connectivity independent of specific stimuli (i.e. faces and words) within movies. For the left amygdala seed term, we saw increased left amygdala-inferior occipital gyrus connectivity as a function of anxiety (peak = [−53 -77 -5], 12 voxels). Following our more liberal, post-hoc thresholding, we also saw increased left amygdala-middle frontal gyrus connectivity (voxelwise *p* < .01, peak = [−41 37 43], 40 voxels) and right amygdala-middle temporal gyrus connectivity (voxelwise *p* < .05, peak = [58–71 10], 195 voxels) as a function of anxiety.

#### 4.4.2 Human Connectome Project

**Fig. 4:**
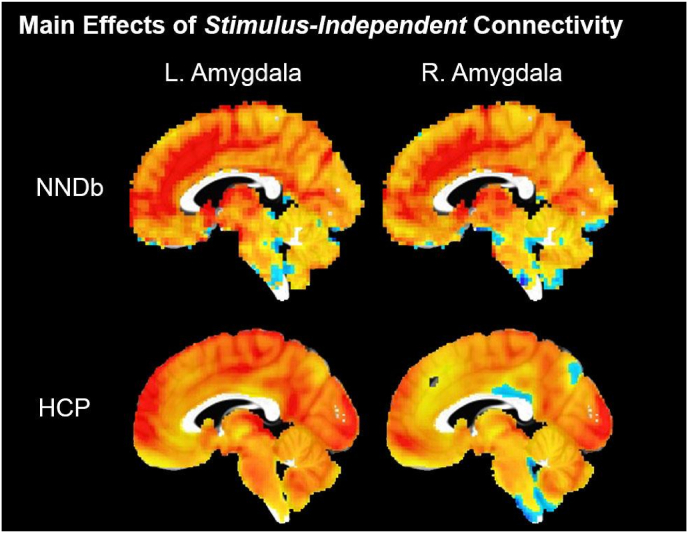
Whole-brain results (*p*_uncorr._<0.001, cluster-corrected) demonstrating main effects of amygdala seed connectivity.

We observed main effects of amygdala seeds consistent with the previous experiment (Fig. 4), including positive connectivity to fusiform face area, prefrontal cortex, and cingulate gyrus. However, we did not observe any corrected correlations between anxiety scores and seed connectivity in whole-brain analyses.

We reconducted the above analyses using Fisher z-transformed correlation coefficients instead of beta-weights. This did not alter our inference (i.e. consistent main effects). Another property of the HCP dataset was that the runs used different phase encoding directions (runs 1/4 = Anterior-Poster, runs 2/3 = Posterior-Anterior). As phase encoding direction is known to have an impact on distortions and signal dropout around the amygdala (De Panfilis and Schwarzbauer, 2005), the variable phase encoding employed in the present dataset could mask results collapsed across runs. As such, we preregistered additional analyses to test effects on anxiety on runs which used Anterior-Posterior and Posterior-Anterior phase encoding separately. For runs with AP phase only (congruent with the Naturalistic Neuroimaging Database) we observed two cluster-corrected (i.e. *p*_uncorr._ < .05, k < 1913.7), anxiety-relevant results: a heightening of left amygdala-right fusiform/cerebellum connectivity (peak = [36–59 -46], 2043 voxels) and a degradation of right amygdala-right fusiform/cerebellum connectivity (peak = [37–78 -19.2], 1950 voxels). Neither of these clusters were apparent in runs which used PA phase (for comparisons of main effects between phases see supplement 4).

### 4.5 Control analyses

For our calcarine connectivity control analysis, we did not find any correlations with anxiety across all voxelwise thresholds (0.001, 0.01, 0.05) in both the Naturalistic Neuroimaging Database and Human Connectome Project. This suggested our previous anxiety-relevant connectivity results were not driven by global noise (e.g. motion; though this assumes between-subject differences in BOLD artifacts are consistent across the whole brain).

### 4.6 Clip-level analysis

**Fig. 5:**
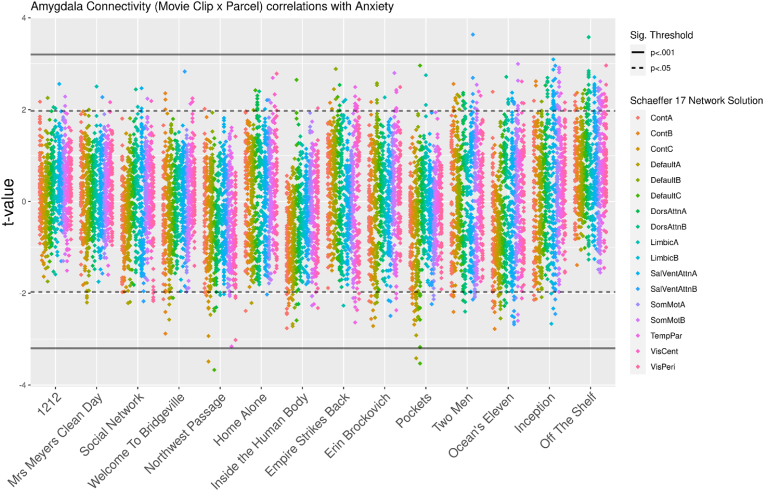
L/R amygdala connectivity (2 × 400 parcels) x anxiety t-scores per movie clip. Clips (x axis) ordered by number of amygdala-parcels demonstrating uncorrected significance (*p* < .05).

For our clip-level analysis, we did not submit clip by parcel model outputs to any formal statistical testing. For descriptive, exploratory purposes only: we note variability in the number of parcels surpassing uncorrected significant thresholds across the clips (range = 7:72; Table 4; Fig. 5).

**Table 4:** Movie clips and the number of amygdala-parcel x anxiety correlations surpassing uncorrected p < .05.

Clip	No. Parcels <.05	Clip	No. Parcels <.05
1212	7	Empire Strikes Back	28
Mrs Meyers Clean Day	7	Erin Brockovich	34
Social Network	14	Pockets	43
Welcome to Bridgeville	16	Two Men	44
Northwest Passage	24	Ocean's Eleven	49
Home Alone	25	Inception	67
Inside the Human Body	27	Off the Shelf	72

## 5 Discussion

This project was motivated by an amygdala-prefrontal model of threat-processing. Initially evidenced from rodent literature (see Robinson et al., 2019), this model outlines an excitatory amygdala-prefrontal circuit which drives harm avoidance (Robinson et al., 2014). fMRI work has implicated a homologous circuit in humans: experiments have demonstrated amygdala-prefrontal coupling to faces appears increased whilst under threat-of-shock, the degree of which correlates with self-reported anxiety (Robinson et al., 2012). In the present preregistered two-experiment study, we sought to extend this model of anxiety to naturalistic settings through means of movie fMRI. To this end, we correlated face-dependent connectivity with self-reported anxiety symptoms in a movie-watching database. In our original analyses, stimulus independent tests suggested self-reported anxiety to correlate with degraded amygdala-anterior cingulate coupling, but only when using post-hoc thresholding. However, we failed to replicate this effect in a second dataset. Moreover, this effect dissipated when using an updated version of the database with improved preprocessing, thus we do not infer this to be a stable finding. Following reanalysis in the updated database, we observed anxiety-relevant correlations with stimulus-onset activation, but did not observe robust alterations in connectivity.

Within our main effects tests of whole-brain activation (experiment one), we report expected engagement of fusiform gyrus and auditory cortex to faces and spoken words respectively. In addition to replicating previously observed effects, this mitigated concerns regarding the accuracy of our stimulus-detection algorithm to adequately detect face and spoken word onset information. Moreover, we noted two correlations with anxiety. As a function of anxiety, we saw greater face-dependent bilateral superior parietal activation and reduced spoken word-dependent activation in left auditory cortex. As these were not hypothesized clusters, we do not comment on these further, but—given that these effects passed our a priori thresholding—future work should seek to test whether these effects are apparent in an independent sample.

For our psychophysiological interaction analyses, we observed widespread main effects. This included increased face-dependent connectivity to inferior frontal gyri, medial prefrontal cortex, and superior temporal gyri. We did not see correlations between anxiety and connectivity in our hypothesized regions, though inspection of results with post-hoc thresholding implicated increased face-dependent amygdala-superior temporal sulcus connectivity as a function of anxiety. We also conducted stimulus-independent analyses across two datasets. We did not observe any cluster-corrected results at our preregistered voxelwise threshold. Using post-hoc thresholding we noted anxiety-relevant amygdala-middle frontal and -middle temporal connectivity in experiment one, and amygdala-fusiform connectivity in experiment two. Given the lack of overlap between these studies and the use of post-hoc thresholding, we do not make a strong inference regarding these. We emphasize however that differences in movie content and length between these two datasets should be considered for future studies wishing to provide replications and/or out-of-sample validation.

### 5.1 Future of anxiety and movie fMRI research

Across the psychological sciences, our theories and models are built on the foundations of highly controlled studies (Yarkoni and Westfall, 2017). Said experimental designs were driven by the need for adequately controlling potential confounds. However, this comes with the cost of limited contextual generalizability. Indeed, our present results highlight a discrepancy when utilizing relatively more naturalistic stimuli. It may be that harm avoidance circuitry is not maximally engaged during face perception. Instead, said processing may occur more broadly for generally salient information in the environment (though our stimulus-independent analyses did not evidence this). Our work has further emphasized the need within affective neuroscience to scrutinize what components of our theory do and don't extend to ecologically-richer settings.

While it has become apparent that movie fMRI can evoke relatively more stable, richer, and clinically insightful functional networks (Meer et al., 2020; Finn and Bandettini, 2020; Eickhoff et al., 2020), the present study highlights the need for careful consideration of stimulus complexity when modelling dynamic movie fMRI data. We were unable to explore the temporal properties (e.g. emotional content) with the data available. However, when we re-analyzed data on a scene-by-scene basis, the results implied that differences may occur as a function of movie stimulus. Given that individual differences in anxiety may be most prominent within threatening environments, directly modelling dynamic, canonical valence/arousal ratings may increase sensitivity to these effects (as has been demonstrated within the depression literature: Gruskin et al., 2020). Moreover, said dynamics may be nested throughout multiple features of the movies, ranging from overall emotional tension to specific content within faces (e.g. novelty, expression).

Alternatively, traditional approaches to fMRI analyses (i.e. feature-based regression) may be particularly limited when attempting to capture anxiety-relevant neural systems during movie-watching. One possible avenue for future work would be to bridge data- and hypothesis-driven approaches through the use of techniques such as intersubject representational similarity analysis (Chen et al., 2020). This may help implicate whether previously reported anxiety-relevant brain circuitry is engaged during movie-watching without the need for assumptions regarding stimulus features or hemodynamic response.

We also highlight here the tools used to assess anxiety in the present project. Though the NIH toolbox offers a useful battery for a wide assessment of cognitive/affective domains, this was a computerized adaptive questionnaire that typically administers far fewer questions than more standardized anxiety questionnaires, such as the state-trait anxiety inventory (Spielberger, 1983), which may be more appropriate for detecting subtle differences along the continuum of anxiety severity. It may also be plausible that the two dimensions of state vs trait anxiety may reveal dissociable effects, though we have previously noted these two measures (as assessed by questionnaires) correlate very highly (r = .83; see Kirk et al., 2021). Consequently, the dissociation of these may be further elucidated through correlations with both questionnaires and regressors marking tonal shifts throughout movie stimuli. We also note the non-clinical nature of the present project. Given that individuals demonstrating particularly high anxiety may avoid volunteering for fMRI studies (Charpentier et al., 2021), explicit comparisons between individuals with anxiety disorders and healthy controls may reveal differences not apparent here.

Finally, we highlight that several of our results presented here were detected using post-hoc voxelwise thresholding. As such, conclusions regarding these effects should be tentative. Furthermore, we also note that our results indicate preprocessing steps (*experiment 1* v1 vs v2) and scanning parameters (*experiment 2*) likely impact the sensitivity of detecting effects of anxiety. Future work interested in investigating amygdala-prefrontal connectivity in movie fMRI should pay particular attention to how the sensitivity of the BOLD signal in medial temporal lobe and prefrontal cortices may be impacted by preprocessing and sequence parameters. Given this limitation, it is not possible within the constraints of the present project to rule out the role of this circuitry in anxiety-related face processing. Nonetheless, we believe the present work has laid foundations to help guide future movie fMRI work into anxiety.

## 6 Conclusion

Our project aimed to test whether an amygdala-prefrontal threat-processing model of anxiety could extend to naturalistic stimuli. We noted effects of anxiety on face-dependent superior parietal activation and word-dependent auditory cortex activation. However, we failed to find a correlation between face-dependent amygdala-prefrontal coupling during movie-watching and self-reported anxiety. Seed analyses also did not reveal robust effects of anxiety-relevant amygdala-cingulate connectivity. Overall, this work tempers the proposed role of this circuitry in anxiety and highlights the importance of testing predictions derived from experimentally constrained contexts in more naturalistic settings to ensure generalizability.

## Credit statement

Conceptualization, formal analyses, and writing of the original draft was conducted by P.A.K. Resources (Naturalistic Neuroimaging Database) were provided by J.I.S. All authors were involved in reviewing and editing the manuscript. Project was jointly supervised by O.J.R. and J.I.S.
