# Supplementary material for: Anxiety and amygdala connectivity during movie-watching
Source: Neuropsychologia. 2022 May 3;169:108194. doi: 10.1016/j.neuropsychologia.2022.108194 (PMC8987737; doi:10.1016/j.neuropsychologia.2022.108194)
Supplement: Multimedia component 1 [file mmc1.docx]

**Supplementary Materials**

**Anxiety and Amygdala Connectivity During Movie-watching**

**By Peter A. Kirk, Oliver J. Robinson, & Jeremy I. Skipper**

**Supplement 1. Age distribution**

Density plot of age for participants in the Naturalistic Neuroimaging Database.

**
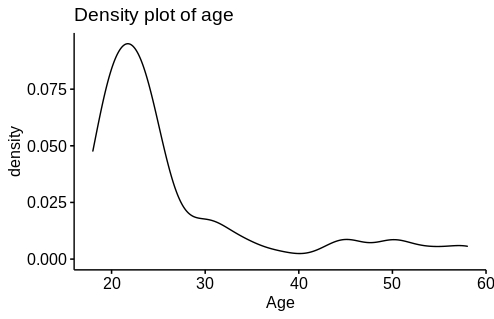
**

**Supplement 2. Regions of Interest analysis (NNDb)**

As whole-brain statistical correction could be overly conservative, we conducted a series of ROI analyses to test our hypotheses in the Naturalistic Neuroimaging Database. We originally preregistered ROI analyses using amygdala beta-weights for face onsets. However, as amygdala seed time series were regressed out in the within-subject models, this yielded beta weights near-zero. As such, we have omitted this from our ROI analyses.

An ANCOVA of dmPFC ROIs on the left amygdala face interaction beta weights demonstrates no main effects of anxiety (F(1,64)=1.25, *p* =.27) or movie (F(9,64)=1.46, *p* =.18). The winning model of the Bayesian ANCOVA on left amygdala face interaction betas was the null model (BF_01_= 1), which was anecdotally better than the anxiety (BF_01_=1.65) and age + anxiety models (BF_01_=2.23), and at least substantially (>7 times) better than all other models including anxiety (BF_01_>7.80).

An ANCOVA of dmPFC ROIs on the right amygdala face interaction beta weights demonstrates no main effects of anxiety (F(1,64)=.53, *p* =.47) or movie (F(9,64)=.68, *p* =.73). The winning model of the Bayesian ANCOVA on left amygdala face interaction betas was the null model (BF_01_= 1), which was substantially better than the anxiety (BF_01_=3.18) and age + anxiety models (BF_01_=9.11), and at least stronger (>10 times) than all other models including anxiety (BF_01_>12.02).

**Supplement 3. Human Connectome Project grey matter masks.**

To derive grey matter masks, we used AFNI’s 3dMean to output only voxels which shared overlap between all participants (‘-mask_inter’). However, this resulted in an overly thin group grey matter map, potentially masking widely shared voxels (below; top row). As such, we used 3dmask_tool to generate a grey matter output which required 95% overlap across participants.


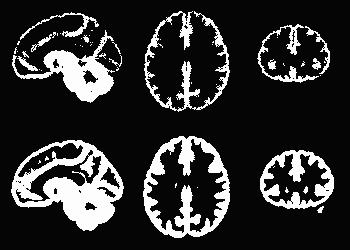


**Supplement 4. Stimulus-independent main effects for runs with AP phase vs PA phase (no thresholding).**

To demonstrate the potential impact of phase encoding on connectivity results from the Human Connectome Project data, we visualized the main effects of stimulus-independent connectivity during movie-watching (though we note these are confounded with between-run differences in movie stimuli).

**
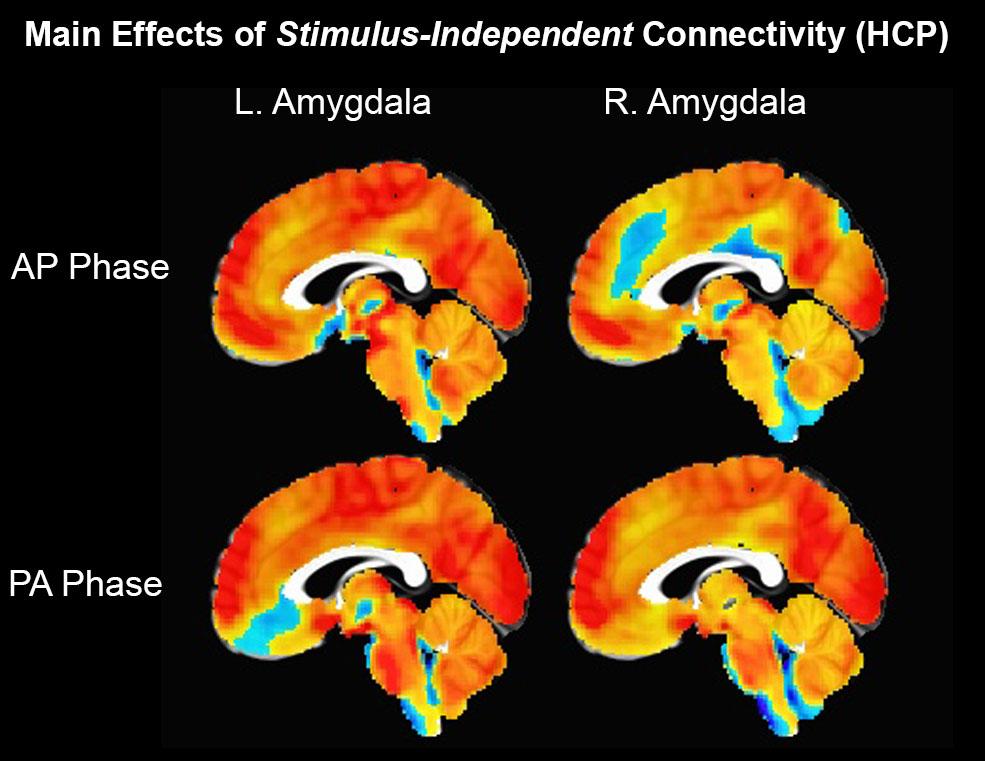
**
